# Association of nutritional status, frailty, and rectus femoris muscle thickness measured by ultrasound and weaning in critically ill elderly patients

**DOI:** 10.5578/tt.20239901

**Published:** 2023-03-10

**Authors:** B. Er, B. Mızrak, A. Aydemir, S. Binay, C. Doğu, D. Kazancı, S. Turan

**Affiliations:** 1 Intensive Care Unit, University of Health Sciences Ankara City Hospital, Ankara, Türkiye; 2 Department of Anaesthesiology and Reanimation, University of Health Sciences Ankara City Hospital, Ankara, Türkiye

**Keywords:** older age, nutrition, screening, ultrasound, intensive care

## Abstract

**ABSTRACT:**

Association of nutritional status, frailty, and rectus femoris muscle thickness measured by ultrasound and weaning in critically ill elderly patients

**Introduction:**

Sarcopenia and frailty are critical factors linked with poor clinical outcomes among elderly individuals. This study aims to investigate the association between nutritional assessment tests and frailty with muscle thickness measured by ultrasound and their relationship with weaning among crtically ill elderly patients.

**Materials and Methods:**

Patients who were over 65 years old and required invasive ventilation were assessed for nutritional status and clinical frailty scale upon admission to the intensive care unit. Additionally, the thickness of their rectus femoris and vastus intermedius muscles were measured by ultrasound within 48 hours of intubation. Correlation analysis was conducted to examine the relationship between screening tests, frailty, and ultrasound results. The association between these parameters and weaning success was also evaluated.

**Results:**

Between May and August 2022, 32 consecutive patients were enrolled in the study. The mean age was 79.3 ∓ 7.9, and 18 (56.3%) of them were female. Median APACHE-II- and first-day SOFA scores were 22.5 (16.2-29.7) and 7 (5-10.75), respectively. There was a moderate negative correlation between the thickness of the rectus femoris and frailty (r= -0.41, p= 0.036), and there was a moderate positive correlation between the rectus femoris and geriatric nutritional risk index (r= 0.45, p= 0.017). Of them, 18 (56.3%) patients were classified as weaning failure in which the mean frailty score was higher (7.6 ∓ 0.9 vs 6.5 ∓ 1.7, p= 0.035), sepsis (18 vs 7, p< 0.001) and use of vasopressor (17 vs 6, p= 0.004) more common, and in-hospital mortality were higher (18 vs 5, p< 0.001).

**Conclusion:**

Bedside ultrasound could be beneficial for detecting nutritional high-risk patients. Frailty was associated with muscle thickness, and it was also associated with weaning failure.

## Abstract (tr)

**ÖZ:**

İleri yaş yoğun bakım hastalarında geriatrik nütrisyonel risk indeksi, kırılganlık ve ultrasonla ölçülen rektus femoris kas kalınlığı ilişkisi ve bunların mekanik ventilasyondan ayrılma süreciyle ilişkisi

**Giriş:**

Sarkopeni ve kırılganlık, yaşlı popülasyonlarda kötü klinik sonuçlarla ilişkilidir. Bu çalışmada ultrasonla ölçülen kas kalınlığı ile kırılganlık ve nütrisyon değerlendirme testleri arasındaki ilişkinin ve bu verilerin hastalarda mekanik ventilatörden ayrılma durumuyla ilişkisinin belirlenmesi amaçlandı.

**Materyal ve Metod:**

Altmış beş yaş üstü invaziv ventilasyon uygulanan hastalar, yoğun bakıma kabul esnasında beslenme değerlendirme testleri ve klinik kırılganlık skalasıyla değerlendirildi ve entübasyonun ilk 48 saatinde ultrason ile rektus femoris ve vastus intermedius kalınlığı ölçüldü. Tarama testleri, kırılganlık ve ultrason sonuçları için korelasyon analizi yapıldı. Bu parametrelerle ventilatörden ayrılma durumunun ilişkisi değerlendirildi.

**Bulgular:**

Mayıs-Ağustos 2022 tarihleri arasında ardışık 32 hasta çalışmaya alındı. Yaş ortalaması 79,3 ± 7,9 olup, 18’i (%56,3) kadındı. Ortanca APACHE-II ve birinci gün SOFA skorları sırasıyla 22,5 (16,2-29,7) ve 7 (5-10,75) idi. Rektus kalınlığı ile kırılganlık arasında orta düzeyde negatif korelasyon (r= -0,41, p= 0,036), rektus kalınlığı ile geriatrik beslenme risk indeksi arasında orta düzeyde pozitif korelasyon vardı (r= 0,45, p= 0,017). Ortalama kırılganlık skorunun daha yüksek olduğu (7,6 ± 0,9 vs 6,5 ± 1,7, p= 0,035), sepsis (18’e karşı 7, p< 0,001) ve vazopresör kullanımının (17'ye karşı 6, p= 0,004) daha fazla olduğu ve hastane mortalitesinin daha yüksek (18’e karşı 5, p< 0,001) olduğu 18 (%56,3) hasta weaning başarısızlığı olarak sınıflandırıldı.

**Sonuç:**

Yatak başı ultrason beslenme açısından yüksek riskli hastaların saptanmasında katkı sağlayabilir. Kırılganlık, yoğun bakıma kabul anında ölçülen rektus femoris kas kalınlığı ile korele, aynı zamanda mekanik ventilatörden ayrılma başarısı ile ilişkilidir.

## Introduction

Elderly patients in intensive care units (ICUs) often suffer from multiple chronic diseases, and in addition to multimorbidity, outcomes can be affected by sepsis, immobilization, and acute illnesses ( 1 , 2). The baseline nutritional status of critically ill patients is a crucial determinant of several outcomes ( 3).

Sarcopenia is defined as progressive loss of muscle mass and function, which is related to worse outcomes like frailty, and mortality ( 4).

Frailty is an emerging syndrome in the elderly population that is associated with poor outcomes and is often defined as a coexisting entity with sarcopenia( 5 ). Several screening tools are available to assess preexisting malnutrition and nutritional status. The modified nutrition risk in critically ill (mNUTRIC) is one of the recommended screening tools according to the guidelines of ASPEN/Society of critical care medicine ( 6 ).

Muscle mass is one of the sarcopenia criteria, and ultrasonography is a bedside applicable noninvasive technique for assessing skeletal muscle mass ( 7 ). In a geriatric population outside of the ICU, it has been found that the thickness of the rectus femoris (RF) muscle is correlated with some nutritional assessment tests ( 8 ).

The association of RF and vastus intermedius (VI) muscle mass with mortality and weaning failure was shown in adult critically ill patients previously( 9 , 10 ). In elderly patients, studies investigating the association of muscle mass measured with ultrasound and weaning outcomes are limited. In this study, we aimed to determine the association between nutritional risk assessment tests and lower limb muscle thickness measured by ultrasound and the association of these parameters with weaning in elderly critically ill patients.

## MATERIALS and METHODS

This study was conducted at Ankara City Hospital, in general ICU. Intubated patients over the age of 65 were consecutively included in the study between May and August 2022. At ICU admission, patients who were intubated more than 48 hours, or had an intubation history in the last six months were excluded. For each patient, the following data were recorded: age, sex, comorbidities, body mass index (BMI), clinical frailty scale (CFS), the reason for ICU admission, The Acute Physiology and Chronic Health Evaluation (APACHE)-II score within 24 hours of admission, and the first-day Sequential Organ Failure Assessment (SOFA) score. Additionally, clinical conditions that occurred during the ICU stay (such as sepsis, acute respiratory distress syndrome, and renal replacement therapy), length of ICU stay, and mortality data were also recorded.

We screened malnutrition using the geriatric nutritional risk index (GNRI), modified Nutrition risk in critically ill (mNUTRIC) score. The GNRI is a simple tool based on the serum albumin level and the ratio of present body weight to ideal body weight which could reflect long-term nutritional status. A spontaneous breathing trial (SBT) was done by the clinician’s decision. Patients were extubated if SBT was tolerated (respiratory rate <35 breaths=""/min, heart rate <140 beats/min, oxygen saturation ≥90%, 80 mmHg< systolic blood pressure <180 <20% change from baseline and absence of increased breathing work or distress signs). Weaning failure was defined as the need for reintubation or mortality within 48 hours after extubation. The patients were categorized into success or failure groups based on these criteria.

The study protocol was approved by the Ethics Committee of Ankara City Hospital (Date: 07/09/2022, Decision no: E2-22-2363). Informed consent was not required as this was a retrospective study.

### Ultrasound measurement

**Figure 1:**
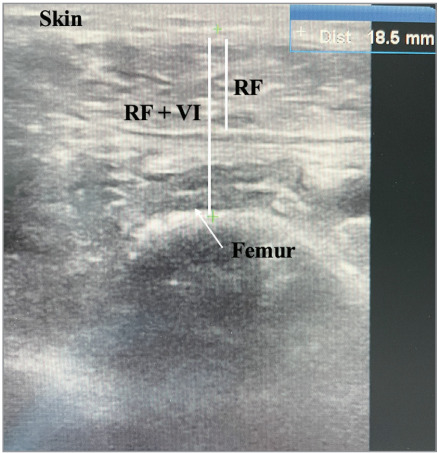
Ultrasound view of rectus femoris (RF) and vastus intermedius (VI) muscles in B mode.

We utilized a 13 MHz high-frequency linear probe (Mindray M7, China) for the measurements. To avoid interoperator variability, a single blinded individual performed all measurements without knowledge of the patients’ clinical status. Measurements were taken on the right leg, with the probe placed perpendicular to the long axis of the muscle at 3/5 of the distance between the anterior superior iliac spine and the superior border of the patella for the thickness of RF and VI, as previously described ( 11 ). Consistent visualization was obtained while the patients were in a supine position with both legs passively extended. Three consecutive measurements were taken, and quantitative parameters were recorded after freezing the ultrasound image ( Figure 1 ).

### Statistics

The statistical analysis was performed using the statistical software package SPSS 23.0.0.2.

The median and interquartile range (IQR) were used for non-normally distributed data, while percentages were used for categorical variables. The patients were classified into two groups based on the weaning outcome: success and failure.

Continuous variables were compared using MannWhitney U test, Fisher’s exact test, and the Chisquare test for categorical comparisons. Spearman test used for correlation analysis in RF and CFS, mNUTRIC, BMI, GNRI. Statistical significance was set at 2-sided p< 0.05 for all the above-mentioned analyses.

## RESULTS

A total of 32 patients were consecutively enrolled in the study; 18 (56.3%) were female, and the mean age (standard deviation) was 79.3 ± 7.9. Clinical features were presented in ( Table 1 ). Hypertension, diabetes, and congestive heart failure were the most common comorbidities. The most common reason for ICU admission was coma which was followed by acute respiratory failure.

Spearman correlation analyses were performed to assess the association between RF alone and with VI, as well as BMI, CFS, mNUTRIC, and GNRI. There was a moderate negative correlation between the thickness of RF and CFS (r= -0.41, p= 0.036), and there was a moderate positive correlation between RF and GNRI (r= 0.45, p= 0.017). Other results are presented in ( Table 2 ).

The patients were divided into two groups based on their weaning outcomes: those who were successfully weaned and those who experienced weaning failure, which was defined as reintubation or mortality within 48 hours after extubation. 18 (56.3%) of them were in the weaning failure group. Demographic, clinical features, and nutritional risk assessment test results were similar in the two groups. The mean CFS was higher (7.6 ± 0.9 vs 6.5 ± 1.7, p= 0.035), sepsis and use of vasopressor during ICU stay were more common, and mortality was higher in the weaning failure group ( Table 3 ).

**Table 1:** Clinical features of the patients (n= 32)

Mean age (SD)	79.3 (7.9)	BMI, kg/m2, mean (SD)	26.1 (5.4)
Female sex, n (%)	18 (56.3)	Reason of ICU admission, n (%)	
Comorbidities, n (%)		Coma	20 (62.5)
Hypertension	22 (68.8)	Acute respiratory failure	18 (56.3)
Diabetes	11 (34.4)	Sepsis	9 (28.1)
Congestive heart failure	11 (34.4)	APACHE-II, median (IQR)	22.5 (16.2-29.7)
Coronary artery disease	10 (31.3)	SOFA, median (IQR)	SOFA, median (IQR)
Chronic obstructive lung disease	10 (31.3)	Length of ICU stay, median (IQR)	22.5 (10-36)
Solid malignancy	7 (21.9)		

n: Number, SD: Standard deviation, IQR: Interquartile range, BMI: Body mass index, ICU: Intensive care unit, APACHE: The Acute Physiology and Chronic Health Evaluation, SOFA: The Sequential Organ Failure Assessment.

**Table 2:** Correlation of screening tests and muscle mass

RF muscle thickness	RF and VI muscle thickness
	rho	p	rho	p
BMI	0.35	0.74	0.36	0.07
CFS	-0.41	0.036	-0.30	0.13
mNUTRIC	-0.04	0.87	-0.009	0.96
GNRI	0.45	0.017	0.38	0.051

RF: Rectus femoris, VI: Vastus intermedius, BMI: Body mass index, CFS: Clinical frailty scale, mNUTRIC: Modified nutrition risk in critically ill, GNRI: Geriatric nutritional risk index, rho: Spearman correlation coefficient.

**Table 3:** Comparison of weaning success and failure groups

Weaning success (n= 14)	Weaning failure (n= 18)	p
Age, mean ± SD	80.8 ± 8.1	78.2 ± 7.8	0.36
Female sex, n (%)	7 (50)	11 (61.1)	0.72
BMI, mean ± SD	25.7 ± 3.7	26.5 ± 6.4	0.67
CFS, mean ± SD	6.5 ± 1.7	7.6 ± 0.9	0.035
mNUTRIC, mean ± SD	6.1 ± 2.4	7.2 ± 2.3	0.23
GNRI, mean ± SD	90.5 ± 14.9	93.7 ± 15.8	0.57
APACHE-II, median (IQR)	19 (13.7-30.5)	24.5 (17.7-29.2)	0.30
SOFA, median (IQR)	6.5 (3-8.2)	7.5 (5.7-12)	0.12
Sepsis, n (%)	7 (50)	18 (100)	<0.001
Vasopressor use, n (%)	6 (42.9)	17 (94.4)	0.004
ARDS, n (%)	0	1 (5.6)	>0.99
RRT, n (%)	3 (21.4)	8 (44.4)	0.27
In-hospital mortality, n (%)	5 (35.7)	18 (100)	<0.001
RF, median (IQR)	9.6 (6.8-13.1)	9.4 (6.6-12.3)	0.67
RF+VI, median (IQR)	19.8 (13.9-28.6)	18 (13.1-24.4)	0.64

n: Number, SD: Standard deviation, BMI: Body mass index, CFS: Clinical frailty scale, mNUTRIC: Modified nutrition risk in critically ill, GNRI: Geriatric nutritional risk index, APACHE: The Acute Physiology and Chronic Health Evaluation, SOFA: The Sequential Organ Failure Assessment, ARDS: Acute respiratory distress syndrome, RRT: Renal replacement therapy, RF: Rectus femoris, VI: Vastus intermedius.

## DISCUSSION

In this study, we found that there was a correlation between the thickness of the RF muscle measured by ultrasound and both GNRI and frailty in the elderly population in the ICU. Additionally, we observed a higher prevalence of frailty in the weaning failure group, although no similar association was observed in other measurements.

For detecting sarcopenia, assessment of skeletal muscles by ultrasound is one of the validated tools ( 7 ). Ultrasound was reported as a reliable and valid tool for the assessment of RF and vastus lateralis with the highest intraclass correlation coefficient scores in older adults ( 12 ).

A cross-sectional study found that RF muscle thickness measured by ultrasound had a high discriminatory power (area under the curve of 0.9) in distinguishing sarcopenia from non-sarcopenia, suggesting the use of ultrasound measurement for screening sarcopenia ( 13 ). In our study, the thickness of RF correlated with GNRI as a risk assessment test, but we did not detect similar results with BMI and mNUTRIC scores. This could be associated with the entity entitled sarcopenic obesity which is reflecting body composition changes occurring with the aging process in the literature ( 14 ).

Frailty was described as a state of vulnerability to poor resolution of homeostasis after a stressor event and is a consequence of the cumulative decline in many physiological systems during a lifetime ( 15 ). In critically-ill patients, frailty was associated with higher hospital mortality, long-term mortality, and several worse outcomes ( 16 ). Consistent with the literature, in our study, the weaning failure group had a higher CFS average. The Clinical Frailty Scale (CFS) has been validated for assessing frailty in the elderly population, however, there is currently no gold standard for its diagnosis. Skeletal muscle evaluation with ultrasound is suggested as a method that can contribute to the diagnosis of frailty, and our results support this with a negative correlation between muscle thickness and CFS ( 17 ).

In the general ICU population, the relationship between RF muscle thickness and weaning success has been investigated before, but there is insufficient data on this subject in the elderly ( 18 ). In our study, although numerically the RF muscle thickness was higher in the successful weaning group, it did not reach statistical significance. As mentioned above, body and muscle composition can change throughout the aging process, and it can limit the value of muscle mass measurement.

This study was conducted in a single center and in a severe patient population which could limit its generalization to the general ICU population. Muscle thickness was evaluated with ultrasound instead of a gold standard technique such as computed tomography. However, it is a validated, noninvasive, and bedside-applicable technique in the ICU.

## CONCLUSION

In conclusion, our study highlights the importance of frailty as a predictor of weaning failure in critically ill elderly patients. Furthermore, we found a significant association between RF muscle thickness and GNRI as well as frailty, suggesting that bedside ultrasound can be a useful tool for identifying nutritional highrisk patients who may benefit from aggressive and multidisciplinary nutritional and rehabilitative interventions.

## Acknowledgment

This research did not receive any specific grant from funding agencies in the public, commercial, or notfor-profit sectors.

## Ethical Committee Approval

The study protocol was approved by the Ethics Committee of Ankara City Hospital (Date: 07/09/2022, Decision no: E2-22- 2363). Informed consent was not required as this was a retrospective study.

## CONFLICT of INTEREST

The authors declare that they have no conflict of interest.

## AUTHORSHIP CONTRIBUTIONS

Concept/Design: All of authors

Analysis/Interpretation: BE, BM, AA, SB

Data acqusition: BE, BM, AA, SB

Writing: BE

Clinical Revision: BE, CD, DK, ST

Final Approval: All of authors

## References

[bb0005] Rudd K.E., Johnson S.C., Agesa K.M., Shackelford K.A., Tsoi D., Kievlan D.R. (2020). Global, regional, and national sepsis incidence and mortality, 1990–2017: analysis for the Global Burden of Disease Study.

[bb0010] Salive M.E. (2013). Multimorbidity in older adults.

[bb0015] Lew C.C.H., Wong G.J.Y., Cheung K.P., Fraser R.J.L., Chua A.P., Chong M.F.F. (2019). The association between nutritional
adequacy and 28-day mortality in the critically ill is not
modified by their baseline nutritional status and disease
severity.

[bb0020] Cruz-Jentoft A.J., Sayer A.A. (2019). Sarcopenia.

[bb0025] Gingrich A., Volkert D., Kiesswetter E., Thomanek M., Sieber C.C. (2019). Prevalence and overlap of sarcopenia, frailty, cachexia and malnutrition in older medical inpatients.

[bb0030] Lee Z.Y., Heyland D.K., Kiesswetter D.K., Thomanek M., Sieber C.C. (2019). Determination of nutrition risk and status in critically Ill patients: What are our considerations?.

[bb0035] Cruz-Jentoft A.J., Bahat G., Bauer J., Boirie Y., Bruyere O., Cederholm T. (2019). Sarcopenia: Revised European consensus on definition and diagnosis.

[bb0040] Ozturk A., Koca M., Burkuk S., Unsal P., Dikmeer A., Oytun M.G., Cederholm T. (2022). The role of muscle ultrasound to predict sarcopenia.

[bb0045] Galindo Martin C.A., Ubeda Zelaya R.D.C., Monares Zepeda E., Lescas Mendez O.A. (2018). ROUNDS Studies: Relation of outcomes with nutrition despite severity-round one: Ultrasound muscle measurements in critically Ill adult patients.

[bb0050] Er B., Simsek M., Yildirim M., Halacli B., Ocal S., Ersoy E.O. (2021). Association of baseline diaphragm, rectus femoris and vastus intermedius muscle thickness with weaning from mechanical ventilation.

[bb0055] Galindo Martin C.A., Monares Zepeda E., Lescas Mendez O.A. (2017). Association of baseline diaphragm, rectus femoris and vastus intermedius muscle thickness with weaning from mechanical ventilation.

[bb0060] Nijholt W., Scafoglieri A., Jager-Wittenaar H O.A., Hobbelen J.S.M. (2017). van der Schans CP. The reliability and validity of ultrasound to quantify muscles in older adults: A systematic.

[bb0065] Rustani K., Kundisova L., Capecchi P.L., Nante N., Bicchi M. (2019). Ultrasound measurement of rectus femoris muscle thickness as a quick screening test for sarcopenia assessment.

[bb0070] Batsis J.A., Villareal D.T. (2018). Sarcopenic obesity in older adults: Aetiology, epidemiology and treatment strategies.

[bb0075] Clegg A., Young J., Iliffe S., Rikkert M.O., Rockwood K. (2013). Frailty in elderly people.

[bb0080] Jung C., Guidet B., Flaatten H. (2022). VIP study group. Frailty in intensive care medicine must be measured, interpreted and taken into account!.

[bb0085] Bencivenga L., Picaro F., Ferrante L., Komici K., Ruggiero F., Sepe I. (2022). Muscle ultrasound as imaging domain of frailty.

[bb0090] Xue S., Xu M., Gu X.P., Ma Z.L., Liu YZhang W. (2022). Advances in ultrasound assessment of respiratory muscle function.

